# Supplementary material for: Procedural Outcomes in Patients Treated with Percutaneous Coronary Interventions within Chronic Total Occlusions Stratified by Gender
Source: J Clin Med. 2022 Mar 4;11(5):1419. doi: 10.3390/jcm11051419 (PMC8911020; doi:10.3390/jcm11051419)
Supplement: Supplementary file 1 [file jcm-11-01419-s001.zip › jcm-1595044-supplementary.pdf]

Table S1. Risk factors for periprocedural death in patients treated with percutaneous coronary intervention within chronic total occlusion.

	OR	95% CI	*P*-value	AUC
Age, per year	1.065	1.018-1.109	0.007	0.69
Gender, male vs. female	0.333	0.132-0.839	0.022	0.62
Weight, per kg	0.965	0.939-0.993	0.016	0.66
Diabetes mellitus	3.579	1.412-9.075	0.008	0.65
Previous PCI	0.222	0.073-0.674	0.003	0.67
Kidney disease	6.029	2.145-16.948	0.004	0.61
Killip class	3.888	1.590-7.016	0.008	0.61
Radial access site during angiogram	0.123	0.039-0.387	<0.001	0.74
Femoral access site during angiogram	8.529	2.713-26.813	<0.001	0.74
Radial access site during PCI	0.148	0.049-0.449	<0.001	0.72
Femoral access site during PCI	7.013	2.307-21.318	<0.001	0.72
Radial access site during angiogram or PCI	0.135	0.045-0.412	<0.001	0.73
Femoral access site during angiogram or PCI	6.905	2.272-20.989	0.001	0.72
LMWH during PCI	13.148	5.081-34.020	<0.001	0.67
LMWH during angiogram or PCI	10.536	3.809-29.140	<0.001	0.69
Aspiration thrombectomy during PCI	31.494	7.083-140.026	0.001	0.55
PCI within LMCA	27.753	9.803-78.567	<0.001	0.63
TIMI after PCI	0.610	0.428-0.841	0.002	0.69
TIMI 3 grade flow after PCI	0.145	0.048-0.442	<0.001	0.72
Dissection during angiogram	18.993	2.425-148.759	0.048	0.53

Cardiac arrest during angiogram	72.846	15.325-346.272	<0.001	0.56
Cardiac arrest during PCI	125.126	39.185-399.555	<0.001	0.61
Cardiac arrest during angiogram or PCI	158.266	57.015-439.323	<0.001	0.66

AUC: area under curve; CI: confidence interval; LMWH: low-molecular weight heparin; OR: odds ratio; PCI: percutaneous coronary intervention

Table S2. Risk factors for periprocedural complications in patients treated with percutaneous coronary intervention within chronic total occlusion.

	OR	95% CI	*P*-value	AUC
Gender, male vs. female	0.651	0.512-0.826	<0.001	0.54
Weight, per kg	0.989	0.982-0.996	0.002	0.55
Arterial hypertension	1.507	1.132-2.005	0.003	0.53
Kidney disease	1.766	1.211-2.576	0.006	0.52
Killip class	2.226	1.410-3.231	0.002	0.53
Radial access site during angiogram	0.651	0.472-0.897	0.01	0.54
Femoral access site during angiogram	1.616	1.172-2.227	0.004	0.55
FFR use during angiogram	2.475	(1.141;5.370)	0.042	0.51
Radial access site during PCI	0.567	(0.453;0.711)	<0.001	0.57
Femoral access site during PCI	1.826	(1.456;2.289)	<0.001	0.57
Radial access site during angiogram or PCI	0.566	(0.451;0.710)	<0.001	0.57
Femoral access site during angiogram or PCI	1.858	(1.483;2.329)	<0.001	0.57
Acetyl-salicylic acid during PCI	1.457	(1.156;1.836)	0.002	0.5424
No P2Y12 during PCI	0.692	(0.552;0.868)	0.001	0.5449
Clopidogrel P2Y12 during PCI	1.310	(1.040;1.648)	0.023	0.5314
LMWH during PCI	2.792	(1.955;3.986)	<0.001	0.5357
ASA during angiogram or PCI	1.612	(1.231;2.111)	<0.001	0.5594
Clopidogrel during angiogram or PCI	1.473	(1.120;1.938)	0.005	0.5481
No P2Y12 during angiogram and PCI	0.563	(0.423;0.750)	<0.001	0.5681
LMWH during angiogram or PCI	2.849	(1.966;4.129)	<0.001	0.5563

IVUS use during PCI	1.983	(1.126;3.495)	0.031	0.5102
Aspiration thrombectomy during PCI	6.258	(2.824;13.865)	<0.001	0.5095
IVUS use during angiogram or PCI	1.978	(1.135;3.450)	0.027	0.5187
PCI within proximal RCA	1.307	(1.012;1.688)	0.044	0.5244
PCI within LMCA	8.316	(5.521;12.526)	<0.001	0.5408
PCI within distal LAD	3.067	(1.765;5.327)	<0.001	0.5150
PCI within proximal Circumflex	1.458	(1.037;2.049)	0.037	0.5180
PCI within medial Circumflex	0.525	(0.306;0.900)	0.01	0.5188
Bifurcation	1.521	1.060-2.184	0.03	0.5174
PCI with drug-eluting stent	0.771	0.615-0.967	0.02	0.5318
Implantation of ≥2 stents	1.871	1.442-2.428	<0.001	0.5498
Implanted stent	0.775	0.618-0.973	0.03	0.5307
PCI with drug-eluting balloon	0.702	0.223-2.210	0.52	0.5025
Total amount of contrast	1.002	1.002-1.003	<0.001	0.5721
TIMI grade after PCI	0.820	0.759-0.887	<0.001	0.5804
TIMI 3 grade flow after PCI	0.457	0.365-0.573	<0.001	0.5945

AUC: area under curve; CI: confidence interval; LMWH: low-molecular weight heparin; OR: odds ratio; PCI: percutaneous coronary intervention

Table S3. Predictors of procedural success expressed as TIMI flow grade 3 after the procedure in patients treated with percutaneous coronary intervention within chronic total occlusion.

	OR	95% CI	*P*-value	AUC
Year of PCI	0.975	(0.957-0.993)	0.007	0.51
Age, per year	0.991	(0.988-0.995)	<0.001	0.52
Gender, male vs. female	0.828	(0.764-0.896)	<0.001	0.51
Weight, per kg	0.996	(0.994-0.999)	0.001	0.52
Diabetes mellitus	0.906	(0.838-0.978)	0.012	0.51
Prior myocardial infarction	0.857	(0.800-0.917)	<0.001	0.52
Prior PCI	0.819	(0.764-0.877)	<0.001	0.52
Prior CABG	0.594	(0.527-0.668)	<0.001	0.52
Arterial hypertension	0.869	(0.803-0.939)	<0.001	0.51
Kidney failure	0.738	(0.643-0.847)	<0.001	0.51
Radial access site during angiogram	1.156	(1.043-1.280)	0.006	0.51
Femoral access site during angiogram	0.874	(0.788-0.970)	0.011	0.51
IVUS use during angiogram	1.792	(0.989-3.246)	0.043	0.50
Radial access site during PCI	1.094	(1.019-1.175)	0.013	0.51
Femoral access site during PCI	0.918	(0.855-0.987)	0.020	0.51
Radial access site during angiogram or PCI	1.088	(1.012-1.170)	0.023	0.51
Femoral access during angiogram or PCI	0.919	(0.856-0.988)	0.021	0.51
No P2Y12 during PCI	0.653	(0.608-0.701)	<0.001	0.55
Ticagrelor P2Y12 during PCI	1.937	(1.592-2.356)	<0.001	0.51
Prasugrel P2Y12 during PCI	1.944	(0.968-3.905)	0.047	0.50

Clopidogrel P2Y12 during PCI	1.399	(1.300-1.506)	<0.001	0.54
UFH during PCI	1.158	(1.050-1.277)	0.004	0.59
Clopidogrel during angiogram or PCI	1.431	(1.318-1.554)	<0.001	0.54
Prasugrel during angiogram or PCI	1.905	(0.948-3.828)	0.055	0.50
Ticagrelor during angiogram or PCI	1.918	(1.573-2.339)	<0.001	0.52
No P2Y12 during angiogram and PCI	0.621	(0.573-0.674)	<0.001	0.56
UFH during angiogram or PCI	1.289	(1.124-1.479)	<0.001	0.51
IVUS during PCI	3.468	(2.512-4.787)	<0.001	0.51
OCT during PCI	3.190	(1.577-6.453)	<0.001	0.50
Rotablation during PCI	5.282	(2.985-9.345)	<0.001	0.50
IVUS during angiogram or PCI	3.022	(2.251-4.056)	<0.001	0.52
OCT during angiogram or PCI	3.012	(1.588-5.712)	<0.001	0.50
PCI within right coronary artery	0.816	(0.761-0.874)	<0.001	0.52
PCI within proximal RCA	0.831	(0.766-0.901)	<0.001	0.52
PCI within medial RCA	1.412	(1.295-1.540)	<0.001	0.53
PCI within distal RCA	2.333	(2.036-2.673)	<0.001	0.53
PCI within RPDA	2.957	(1.896-4.610)	<0.001	0.50
PCI within RPAV	2.199	(1.503-3.217)	<0.001	0.50
PCI within left main coronary artery	2.247	(1.584-3.188)	<0.001	0.50
PCI within LAD	1.510	(1.395-1.636)	<0.001	0.54
PCI within proximal LAD	1.639	(1.482-1.813)	<0.001	0.53
PCI within medial LAD	1.596	(1.434-1.777)	<0.001	0.52
PCI within distal LAD	2.681	(1.891-3.800)	<0.001	0.51
PCI within 1st diagonal	2.799	(2.120-3.695)	<0.001	0.51

PCI within circumflex artery	1.154	(1.054-1.265)	0.002	0.51
PCI within proximal circumflex	1.165	(1.032-1.315)	0.013	0.51
PCI within medial circumflex	1.269	(1.116-1.443)	<0.001	0.51
PCI within distal circumflex	1.484	(1.090-2.020)	0.01	0.50
PCI within 1st obtuse marginal	1.405	(1.189-1.661)	<0.001	0.51
PCI within ramus intermedius	1.894	(1.301-2.756)	<0.001	0.50
PCI within saphenous vein graft	0.599	(0.432-0.831)	0.002	0.50
PCI within Bifurcation	2.322	(1.991-2.707)	<0.001	0.52
PCI with drug eluting stent	69.061	(61.594-77.433)	<0.001	0.89
PCI with bioresorbable vascular scaffold	24.481	(6.032-99.359)	<0.001	0.50
PCI with bare metal stent	3.441	(2.275-5.203)	<0.001	0.51
PCI with ≥2 stents	16.507	(13.284-20.511)	<0.001	0.60
Implanted stent	81.155	(72.335-91.051)	<0.001	0.90
PCI with drug eluting balloon	2.614	(1.798-3.800)	<0.001	0.51
No-reflow during PCI	0.097	(0.045-0.206)	<0.001	0.50
Coronary artery perforation during PCI	0.315	(0.210-0.474)	<0.001	0.50
Death during PCI	0.146	(0.048-0.442)	<0.001	0.50

AUC: area under curve; CI: confidence interval; LMWH: low-molecular weight heparin; OR: odds ratio; PCI: percutaneous coronary intervention
